# Changes in articular cartilage thickness of the knee in JIA as assessed by ultrasonography

**DOI:** 10.1186/1546-0096-9-S1-P44

**Published:** 2011-09-14

**Authors:** D Pradsgaard, A Spannow, C Heuck, A Hørlyck, T Herlin

**Affiliations:** 1 Department of Pediatrics, Aarhus University Hospital Skejby, Skejby, Denmark; 2 Department of Radiology, Aarhus University Hospital Skejby, Skejby, Denmark

## Background

Joint cartilage degradation is resulted by ensuing chronic inflammation in juvenile idiopathic arthritis (JIA). Since the primary goals of treatment are to prevent joint destruction sensitive methods are needed to evaluate cartilage thickness (CTh). Ultrasonography (US) is a reliable tool in the assessment of joint cartilage in rheumatic disease. Previously, we have shown that CTh, in healthy children between 7 and 16 years, is significantly influenced by age and gender.

## Aim

To evaluate the influence of JIA subtype, disease duration and activity on knee CTh in JIA.

## Methods

A cross-sectional study on 73 JIA patients: 13 systemic onset, 35 oligoarticular (extended/persistent: 11/24), and 25 polyarticular (RF-/+: 19/6) JIA. The patients were all Danish Caucasians, age mean (range) 10.2 yrs (5-15), boys/girls: 24/49. Knee CTh was measured on the femur at the midline of the intercondylar notch by greyscale US, based on EULAR standard scans. Clinical and laboratory examinations were performed on the same day as US.

## Results

CTh decreased with increasing age (p<0.001). When controlling for age we found significant difference between CTh for boys and girls 3.0 mm (±0.6) vs. 2.5 mm (±0.6), p<0.0001). Degradation of knee CTh was less in oligoarticular JIA than in polyarticular JIA (p<0.001) and systemic onset JIA (p=0.02) when controlling for age and gender. In polyarticular JIA disease duration was negatively correlated to CTh (p=0.011) whereas no relation was found for oligoarticular and systemic JIA, when controlling for age and gender. Patients with history of active arthritis in both knees had significantly lower CTh than patients without (p<0.05), and in specific knees with a history of active arthritis CTh significantly decreased compared to non-active knees (p=0.039), when controlling for age, gender and disease duration. In knees with a history of intraarticular corticosteroid injections CTh was significantly decreased compared to non-injected knees (2.8 mm (±0.7) and 2.6 mm (±0.5), p=0.031), when controlling for age, gender and disease duration.

## Conclusion

In a cross-sectional study we found that the articular CTh of the knee in JIA was influenced by age, gender and subtype. Further we found that a history of active arthritis or corticosteroid injection decreased CTh. Disease duration was negatively correlated to CTh but only in polyarticular JIA.

